# How does perceptions of social justice affect farmers’ political participation?—Evidence from China

**DOI:** 10.1371/journal.pone.0295792

**Published:** 2023-12-20

**Authors:** Zijian He

**Affiliations:** School of Politics and Public Administration, Wuhan University, Wuhan, China; National Technical University of Athens: Ethniko Metsobio Polytechneio, GREECE

## Abstract

Ronald Inglehart’s postmaterialist theory suggests that with the advancement of industrialization and economic prosperity, there will be a significant transformation in people’s societal values. Concurrently, their forms of political participation shift from conventional activities to unconventional politic activities. However, most research on this topic has been predominantly focused on Western countries. In fact, rural farmers in China serve as an excellent experimental group for testing this theory since they have experienced rapid economic growth while still being deeply influenced by traditional authoritarianism culture.Using a sample of 6,689 respondents from the 2019 Chinese Social Survey (CSS) and employing a Binary Logistic Regression Model, we discovered that Chinese farmers’ perception of overall societal justice exhibits a U-shaped relationship with various forms of political participation. Specifically, it shows a significant negative correlation with non-institutional political participation, such as contact-officer participation, but a significant positive correlation with institutional political participation types like community participation and election participation.Our further research indicates that the three subtypes of perception of societal justice are significantly negatively correlated only with non-institutional political participation, while their statistical relationship with institutional political participation is not significant. We believe that the underlying reason for this phenomenon lies in the unique interpretation of societal justice within Chinese traditional culture. Additionally, through a comparative analysis of models on political participation behavior and willingness, we found that despite significant inequalities and disparities in institutional structures and levels of economic development between rural and urban areas in China, rational considerations of the risks and costs associated with defying the government deter Chinese farmers from engaging in non-institutional politic activities unless their emotional resentment towards unjust practices reaches a certain threshold.

## Background

Ronald Inglehart’s theory of "post-materialism" posits that industrialization and economic prosperity drive a gradual shift in traditional values towards secular rationality and self-expression. Concurrently, there is a transformation in people’s modes of participation, moving from conventional political activities to unconventional forms of politic engagement [1–3]. However, research on this subject has predominantly centered around Western countries [4–7], with limited exploration in non-Western nations. Chinese rural farmers emerge as an excellent experimental cohort for this theory, as they have witnessed rapid economic growth while remaining deeply influenced by traditional authoritarianism culture. This makes them a compelling subject within the purview of this theory’s framework.

In this study, the identification of "farmers" is determined based on their affiliation with the "hukou" system. The Hukou system in China is a mandatory household registration system that assigns urban/non-agricultural or rural/agricultural hukou based on an individual’s place of birth. This system benefits urban residents while discriminating against rural residents in accessing state-owned resources such as employment, education, healthcare, and housing [8]. The hukou system restricts the spatial mobility of individuals based on their hukou status, presenting significant barriers for rural residents when relocating to urban areas. The hukou system acts as a metaphorical "Great Wall," dividing urban and rural regions, exacerbating regional inequalities, and is often viewed as China’s version of racial segregation policies [9].Furthermore, since the reforms and opening-up in 1978, China has undergone profound transformations. Industrialization, marketization, and urbanization reforms have brought rapid economic growth but have also led to a sharp increase in societal and economic inequalities [10]. China has achieved unprecedented GDP growth rates, but income inequality has become one of the most severe among countries, reaching a peak Gini coefficient of 0.4910 in 2008 [11]. Chinese scholars assert that China has shifted from being one of the world’s most egalitarian societies to one of the most unequal over the past few decades [10]. In this context, how do farmers respond to the injustices they face?

Based on the theory of relative deprivation, farmers, as a vulnerable group in the process of market reforms in China, are often considered to have benefited very little from the reforms and are more likely to feel anger towards the current injustices resulting from the exacerbated inequality. However, numerous studies have found that over half of rural residents overestimate their actual societal status [12]. Meanwhile, Chinese farmers do not seem to question the legitimacy of the political system as predicted by many [13]. Some attribute this phenomenon to China’s traditional political culture, where outcomes tend to be valued more than how those outcomes are achieved [14]. Additionally, others attribute it to the unique nature of political participation in China compared to democratic states and other authoritarian countries. Under China’s political system characterized by the "party-state" structure [15], different forms of political participation receive differential treatment from Chinese government authorities, resulting in varying degrees of opportunity structures, political risks, and transaction costs. This experience challenges Western scholars’ definitions and standards of political participation.

Based on the aforementioned studies, we posit that the choice of different modes of political participation among Chinese farmers can be attributed to their understanding of the "justice" of the prevailing circumstances, which often diverges from Western conventional thinking. As argued by David Miller [16], the judgment of whether something is fair or unfair depends not only on the justice principles held by individuals but also, to some extent, on the nature of the situation. Additionally, this article suggests that the relationship between the sense of societal justice and political participation is influenced by both emotional and rational factors. On one hand, based on the theory of relative deprivation, individuals tend to vent their anger and resentment through forms of political participation outside established institutions. However, on the other hand, driven by rational considerations, individuals may be reluctant to engage in politically risky activities. It is only when their emotions reach a certain threshold that they resort to extralegal political actions.

## Conceptual discrimination and literature review

Why do some individuals engage in politics while others have little or no involvement? This question has long been one of the most important and enduring issues in the field of political science. Previous research on the determinants of political participation has primarily employed two approaches. The first approach is sociological, focusing on structural-objective variables and emphasizing that individuals with higher socioeconomic status exhibit significantly higher levels of political participation compared to those with lower socioeconomic status [17–20]. The second approach is psychological, concentrating on individual attitude variables [21–24]. Many scholars have examined factors such as political efficacy, political interest, and political knowledge, consistently finding a positive correlation between these attitudes and political participation [25–30]. Attitudes pertain to individuals’ inclination toward participation and are typically assessed in terms of political beliefs, willingness to participate, or potential for engagement [31].

Within the sociological perspective, the dominant framework is rational choice theory, which directs attention to structural-objective variables. According to this approach, individuals are viewed as rational actors driven by self-interest and motivated to maximize their utility. Their decisions to engage in or refrain from political participation are guided by cost-benefit calculations [32]. Rationally inclined individuals will opt out of political participation if the costs outweigh the benefits [33]. Potential voters or protesters encounter various impediments. Firstly, the probability of an individual’s participation in voting or protesting leading to any significant impact is nearly negligible [34]. Secondly, information costs and cognitive limitations constrain many citizens, preventing them from obtaining accurate assessments of the utility associated with participation. Lastly, most of the benefits derived from participation are public goods, enjoyed equally by participants and non-participants, thus enabling free-riding [35]. Consequently, the success of protest movements relies on external support they can harness, while the costs and rewards of engaging in protest activities are contingent upon societal structures [36]. In such circumstances, "shifting political opportunities and constraints" serve as motivators for individuals lacking personal resources to partake in extralegal political activities [37].

In the psychological perspective, the dominant framework is relative deprivation theory. According to Lerner’s "just world hypothesis," individuals have a need to believe in a "just world" where everyone receives what they deserve. When people encounter information that contradicts their belief in a just world, they experience frustration. Individuals can respond to perceived injustice in various ways. Some feel morally outraged and seek to restore justice [38]. Others may express contempt towards the victims [39] or adopt belief systems that help justify existing societal, economic, and political arrangements [40]. Building upon the just world hypothesis, researchers have proposed the "relative deprivation theory" to explain why some individuals engage in political activities while others do not. With the diffusion of technology, the expansion of the internet, and the reach of social media platforms, people are increasingly exposed to idealized portrayals and lifestyles of others that may be disconnected from reality. These disparities can potentially trigger feelings of localized resentment and frustration, prompting individuals to participate in various forms of political activities [41–44].

We believe that the aforementioned theories have their limitations. The just world hypothesis and relative deprivation theory excessively emphasize the role of emotions, while rational choice theory leans towards a more rational perspective. In reality, both emotional and rational factors play a role in individual behavioral choices. From a psychological standpoint, reaching a certain level of relative deprivation can trigger extralegal political protests. However, from an interactive perspective between behavior and societal structures, individuals base their decisions on a comprehensive assessment of personal resources, skills, societal structures, political systems, and other factors. They rationally evaluate the expected outcomes of their actions—if the benefits are lower than the costs, the sense of relative deprivation may not yield practical results. Therefore, political participation is the result of the interplay between emotions and rationality. To integrate these two perspectives, our research focuses on individuals who actively participate in politics as well as those who express a willingness to participate but do not engage. If an individual’s political participation reflects the influence of emotional factors, then their non-participation is more susceptible to rational considerations. Hence, this paper examines individual emotions, suggesting that the perception of societal justice affects political participation, driven by emotional needs. Additionally, we also consider the structural characteristics of the perception of societal justice and political participation, recognizing that societal structures encourage individuals to make rational choices. When people participate in politics due to unjust societal structures, they may find themselves unable to resolve issues or facing increased risks by engaging further. In such cases, the sense of unfairness may not increase the likelihood of their participation; instead, individuals are more likely to adopt an indifferent or observant attitude, influenced by rational considerations of costs and benefits on political participation. In other words, the interaction of demand stimulation and cost-benefit mechanisms creates different, even contradictory relationships between the perception of societal justice and political participation.

## Theoretical basis and research hypothesis

### Perception of society justice

The term "perception of societal justice" in this article can be defined as individuals’ subjective judgments, evaluations, and attitudes towards the distribution of societal resources [45]. It can further be distinguished between macro-level distributive fairness based on societal inequality and micro-level fairness based on individual income distribution [46]. Discussions and analyses of societal justice have long been present in political and social theories and practices, but there are disagreements regarding its precise meaning and implications. Political, legal, and moral philosophers have extensively deliberated on the "nature of justice" [47–49]. Meanwhile, social psychologists strive to understand the subjective experiences of justice or injustice among individuals [50,51]. Some scholars view societal justice as a two-dimensional concept: distributive justice and procedural justice [52]. Distributive justice refers to societal, economic, individual, and regional disparities arising from resource allocation [53]. Procedural justice, on the other hand, pertains to the perceived fairness of societal norms, rules, regulations, and policies used in decision-making that lead to differential and unequal socioeconomic outcomes within society [54]. Additionally, since distributive justice often involves the distribution of societal and economic resources [55,56], it is commonly explained using four principles of justice attitudes: equality, fairness, need, and entitlement [57]. Extensive empirical research from psychology and sociology supports the categorization of these four fundamental principles of distributive justice [58]. Given the diversity in the conceptual and practical implications of societal justice and the unique context of rural China, I adopt David Miller’s theory of societal justice in this paper. Miller [16] found that people’s views on justice are actually multifaceted as they are determined by the context of a situation. This suggests that determining whether something is just or unjust depends not only on the principles of justice individuals hold but also to some extent on the nature of the circumstances.

David Miller has identified three principles of societal justice: Equality, Need, and Desert. Equality refers to equal treatment and protection as stipulated by laws, inclusive and fair political participation, and equal opportunities for positions within political institutions, primarily applicable in the realm of citizenship. In this interpersonal model, "anyone who is a formal member of such a society is understood as a holder of a package of rights and obligations that together define the status of citizenship," with equality being the primary principle of distributing citizenship. However, different individual needs may mean that equal treatment alone cannot guarantee just or equitable outcomes. Therefore, the second principle of societal justice is Need, which suggests that when some individuals have different needs from others, they should receive special consideration. This principle primarily operates within "solidaristic communities" composed of individuals in face-to-face relationships and connected through "mutual understanding and trust." Miller presents the third principle, Desert, which becomes most evident in instrumental associations, particularly when considering remuneration differentials related to work, income, and resource allocation. The key feature of the Desert principle is that in terms of rewards, participants who contribute more may expect to receive more.

In empirical research on societal justice, the notion of macro-level justice pertains to the perception of overall distribution within society, while micro-level justice focuses on individual beliefs about the fairness of their own income [59]. There exists a distinction between these two aspects [60]. For example, when presented with questions such as "Is your income fair?" and "Is the income distribution in society as a whole fair?", survey respondents tend to have a stronger personal stake in the former. Consequently, directing attention towards overall justice may broaden the scope of issues considered by justice researchers and overcome certain limitations present in current justice studies [61]. Hence, this study examines both the micro-level perception of societal justice and the macro-level or overall perception of societal justice simultaneously.

### Political participation

"Political participation serves as a mechanism through which citizens can exchange information regarding their interests, preferences, and needs, generating responsive pressure" [20]. Over the past 80 years, typologies of political participation in democratic countries have been highly debated. Since the 1970s, research on political participation has often classified political actions such as protests, social movements, and demonstrations as "unconventional" or "informal" forms of political engagement [62]. Starting from the massive wave of participation in the 1960s that swept across most Western democratic nations, there has been a significant increase in protest participation outside of traditional channels, to the point where the conventional distinction between traditional and non-traditional participation has become outdated. People prefer to discuss "institutionalized participation" carried out by individual citizens or through political parties and unions, as well as "protest-oriented participation." Scholars argue that these non-institutionalized forms of political participation are better suited to meet the demands of the new generation of citizens [63–65].

Therefore, based on existing literature, this study conceptualizes political participation into two categories: actions that are perceived as politically legitimate and low-risk, which I refer to as "institutionalized" forms of political participation, and actions that are seen as carrying higher risks and are politically illegitimate, which I term as "non-institutionalized" forms of political participation. These two forms of political participation under investigation differ in terms of risk, demands, and the perceived legitimacy of political engagement. Typically, institutionalized political participation activities encompass electoral participation or community involvement, as well as non-institutionalized political participation activities such as participating in demonstrations, engaging in social media activism, contacting officials, and so forth [66]. Different forms of political participation require varying skills, involve different degrees of collaboration with others, attract diverse actors, and impact the political process in distinct ways [67].

Existing research often considers political participation as a continuous latent variable, with various forms of participation representing manifestations of this underlying construct [68–70]. In the context of China specifically, the distinction between "institutionalized" and "non-institutionalized" political participation simply signifies different levels of willingness to take risks associated with potential repercussions from government or public sanctions. Evidently, the government tends to promote and guide political participation through institutionalized and legitimate channels, while non-institutionalized forms of participation are perceived as challenging and threatening by the government [71]. Although some scholars [72–74] recognize the distinction between different forms of political participation as essentially characterized by high-cost and high-risk versus low-cost and low-risk forms, these studies often rely on case studies that are difficult to generalize to broader populations. To address these issues, this study employs a nationally representative dataset to examine the impact of perception of societal justice on both forms of political participation.

Firstly, although senior government officials in China are not directly elected by citizens, the country’s 1982 constitution stipulates that "urban and rural residents belong to grassroots autonomous organizations based on resident committees or villagers’ committees established locally." Village committee members are directly elected by citizens on an annual basis. This institutional arrangement holds historical significance as it allows the public to directly select and supervise their leadership [75–77]. Within the framework of organizational law, the election of village committees by villagers serves as the foundation for grassroots autonomy. It provides villagers with the opportunity to exercise self-governance at the leadership level and participate in village affairs. In urban areas of China, due to the primary allocation of most material and non-material resources being undertaken by people’s work and service units, community power in resource allocation is quite limited. Conversely, in rural areas of China, almost all resource and value distribution for rural residents is facilitated through the villagers’ committee. Thus, "electoral participation" and "community participation" represent the two primary institutionalized forms of political participation for Chinese farmers.Secondly, if participation in grassroots elections and community affairs is a form of institutionalized political participation, then farmers’ protest activities can be considered a non-institutionalized form of political participation. O’Brien uses the concept of "rightful resistance" to describe this struggle in rural areas of China [78]. Many case studies have examined individuals who have participated in protests, but in reality, protesters often constitute a minority [79,80]. Apart from engaging in protests, Chinese farmers typically resort to direct or indirect contact with government officials to raise issues, express opinions, or seek assistance in search of solutions. Existing research indicates that Chinese citizens are more likely to "seek leaders" directly or indirectly (through acquaintances or friends) in political participation. However, when it becomes difficult to effectively resolve issues through such channels, people are likely to resort to action and employ direct, forceful, or subtle forms of struggle and "pressure" to seek solutions. Lastly, it is worth noting that as a new form of political participation, engagement through social media, particularly the internet, is becoming increasingly widespread among Chinese farmers. In the past, the primary media used by Chinese farmers were television, followed by print media and interpersonal communication. With the gradual spread of the internet in rural areas, rural residents find it easier to access information about national policies and societal issues, and engage in public affairs and political discussions.

Therefore, drawing on the academic discourse surrounding typologies of political participation and taking into account the specific institutional environment of Chinese farmers, we construct five types of political participation: Community participation, Election participation, Rights protection participation, Social media participation, and Contact-officer-participation.These types are based primarily on the degrees of institutionalization and risk, supplemented by dimensions such as influence and conflict level, aiming to examine the politics-society characteristics embodied in different forms of political participation. The first two types belong to institutionalized political participation, while the latter three fall under non-institutionalized political participation.

### Research hypothesis

Considering the different types of perception of societal justice, research has found that individuals who perceive higher fairness in elections are more willing to participate in voting activities, while those who perceive lower fairness tend to prefer non-institutionalized political participation [81,82]. Citizens who experience unfair treatment by government employees are more likely to engage in non-institutionalized political participation, whereas citizens who perceive greater income fairness are less likely to engage in non-institutionalized political participation. Additionally, scholars argue that there is a significant correlation between the level of societal security and the occurrence of non-institutionalized political participation, as the absence of societal security policies and inadequate treatment can trigger such participation [83]. These studies demonstrate the impact of perception of societal justice on political participation across domains such as politics, economics, and societal security. Based on this, we propose the following two hypotheses:

Hypothesis 1: The perception of societal justice among Chinese farmers has a significant negative effect on non-institutionalized political participation. The weaker the perception of societal justice among the public, the higher the likelihood of engaging in rights protection participation activities. The same logic applies to non-institutionalized social media participation and contact-officer-participation.Within this context, we propose the following sub-hypotheses:

Hypothesis 1.1: Desert-based social justice among Chinese farmers has a significant negative effect on their non-institutionalized political participation.

Hypothesis 1.2: Need-based social justice among Chinese farmers has a significant negative effect on their non-institutionalized political participation.

Hypothesis 1.3: Equality-based social justice among Chinese farmers has a significant negative effect on their non-institutionalized political participation.

Hypothesis 2: The perception of societal justice among Chinese farmers has a significant positive effect on their institutionalized political participation. In other words, the stronger the perception of societal justice among Chinese farmers, the higher the likelihood of engaging in institutionalized community participation and electoral participation.Within this context, we propose the following sub-hypotheses:

Hypothesis 2.1: Desert-based social justice among Chinese farmers has a significant positive effect on their institutionalized political participation.

Hypothesis 2.2: Need-based social justice among Chinese farmers has a significant positive effect on their institutionalized political participation.

Hypothesis 2.3: Equality-based social justice among Chinese farmers has a significant positive effect on their institutionalized political participation.

While some factors can predict the intention for political participation, there is limited research that provides a strict definition of this concept. Drawing upon existing definitions of "political participation" and utilizing data from the 2019 Chinese Society Survey, this study defines "political participation intention" as the willingness of individuals who do not engage in activities influencing government decisions to participate in activities that influence political decision-making. Rational choice theory suggests that the choices made by most individuals regarding political participation are typically rational. As rational actors, they evaluate costs and risks, prioritize self-protection, and generally refrain from engaging in illegal or unlawful behavior. However, some individuals may express a willingness to participate but fail to take actual action due to their assessment of the associated costs and risks. In such cases, the sense of relative deprivation does not play a motivating role. Conversely, it can be inferred that, under the influence of risk assessment and self-awareness, once a fair societal context emerges, individuals tend to participate in politics. Even if they engage in non-institutional political activities, they need not worry about unfair treatment afterward.Therefore, we propose:

Hypothesis 3: The perception of societal justice among individuals has a significant positive effect on their willingness for political participation. Specifically, individuals with stronger perceptions of societal justice are more inclined to engage in political life, and this inclination for participation further influences their subsequent political participation behaviors.Within this framework, we put forth the following sub-hypotheses:

Hypothesis 3.1: Desert-based social justice among Chinese farmers has a significant positive effect on their willingness for political participation.

Hypothesis 3.2: Need-based social justice among Chinese farmers has a significant positive effect on their willingness for political participation.

Hypothesis 3.3: Equality-based social justice among Chinese farmers has a significant positive effect on their willingness for political participation.

## Methods

Based on the characteristics of the variables, this study employs the binary logistic regression model to examine the relationship between perception of social justice and political participation, conducting robust regression analysis. Additionally, by comparing the models of political participation behavior and political participation intention, we achieve a similar effect to robustness checks. The first part of our research aims to analyze actual political participation behavior in order to reveal the influence of perception of social justice on farmers’ political participation. The second part focuses on farmers’ willingness to engage in political participation to understand the impact of perception of social justice on their motivation and propensity for active political participation. The different designs and analytical methods employed provide a comprehensive perspective for understanding the political participation behavior of Chinese farmers under varying perceptions of social justice. By considering both actual behavior and willingness, researchers can better grasp the relationship between perception of social justice and political participation, thereby providing targeted recommendations for relevant policy-making.

### Data

The study utilized data from the 2019 Chinese Social Survey (CSS), conducted by the Institute of Sociology at the Chinese Academy of Social Sciences. The CSS provides invaluable insights into complex dynamics such as economic development, social inequality, family structure, and cultural transformations. Recognized as an essential tool for studying various aspects of Chinese society, the CSS guides evidence-based policies. In summary, the CSS offers scientifically rigorous data to comprehend social changes in China. Researchers can access the CSS 2019 data by applying through the "China Social Quality Database Website (csqr.cass.cn)." This platform facilitates exploration of the CSS data and contributes to social science research in China. The CSS 2019 survey focused on the theme of "Social Quality and Social Class Transformation." Covering a broad range of topics, including family dynamics, employment patterns, economic status, living conditions, social security, social values and evaluations, social participation, political engagement, and volunteer service, the survey collected extensive data. It comprised 10,283 valid questionnaires from urban and rural households in 596 villages/communities across 149 cities/counties/districts nationwide, resulting in over 11.6 million data points. Based on existing literature and relevant research, we define farmers as Chinese citizens with agricultural household registration. After excluding missing values, a total of 6,689 valid samples were selected for analysis. Through careful exploration, this study thoroughly examines the credibility and reliability of the research findings, providing valuable insights into the relationship between social justice perception and political participation among Chinese farmers.

### Measurements

One of the dependent variables is a measurement of political participation behavior, primarily assessing whether respondents have participated in political activities. As mentioned earlier, it is categorized into five types. "Participating in collective rights protection actions" is referred to as rights protection participation; "expressing societal issues to newspapers, radio, online forums, and other media" corresponds to social media participation; "providing feedback to government departments" represents contact-officer participation; "engaging in discussions on village, organizational, or major decisions" indicates community participation, while "participating in village committee elections" refers to election participation.As for the second dependent variable, the measurement of political participation intention mainly asks respondents if they would be willing to participate in political activities if they haven’t already. It includes the same five types: rights protection participation, social media participation, contact-officer participation, community participation, and election participation.

The independent variables include evaluations of various subtypes of perception of societal justice and an overall assessment of perception of societal justice. In the CSS2019 dataset, evaluations of fairness in eight societal domains are involved. Based on the aforementioned categorization and considering relevant research findings, this study selects seven aspects, excluding the gaokao system. Through reliability testing, the Cronbach’s alpha coefficient for this scale was found to be 0.844, indicating that they can be averaged by perception of societal justice type for data analysis.Regarding the measurement of overall social justice, respondents were primarily asked to evaluate the current overall state of social justice on a scale from 1 to 10, with 1 representing "very unfair" and 10 representing "very fair."

As for control variables, this study controls for individual anthropological characteristics, economic-societal variables, temporal and spatial effects, such as subjective societal status, income, educational attainment, generational effects, and regional effects.

## Results

### Descriptive statistics

Table 1 presents the mean and standard deviation of the dependent variables (rights protection participation, social media participation, contact-officer participation, community participation, and election participation) and independent variables (overall social justice, desert-based social justice, need-based social justice, and equality-based social justice).The average value for overall social justice is 6.68, with a standard deviation of 2.222. This variable represents participants’ assessment of the current state of overall social justice. Desert-based social justice has an average value of 2.967 and a standard deviation of 0.903. It reflects participants’ views on the fairness of wealth and income distribution, as well as employment and job opportunities.Based on need-based social justice, the average value is 2.876, with a standard deviation of 0.781. It pertains to participants’ perspectives on societal welfare, such as public healthcare and elderly care. Equality-based social justice has an average value of 3.21 and a standard deviation of 0.853. It encompasses participants’ evaluations of rights and treatment between urban and rural areas, as well as political rights and the judicial system.The table also includes control variables. Political identity has an average value of 0.07, indicating a relatively low proportion of party members among the participants. The average household income is 10.481 (log-transformed), with a standard deviation of 1.272. The average education level is 1.92, indicating that the majority of participants have completed secondary education. The average subjective societal status is 1.56, suggesting that participants’ subjective societal status is primarily at a moderate level. The average internet usage is 1.41, indicating that most participants do not use the internet extensively. The average age is 46.716 years, with a standard deviation of 14.2. The average gender value is 0.42, indicating a relatively higher representation of males in the sample. The average cohort effect is 4.205, reflecting a mix of different generational cohorts in the study. The average regional effect is 3.56, representing a wide geographical distribution across different regions in China.These statistical summaries provide an overview of the central tendency and dispersion of variables in the dataset used for analysis.

**Table 1:** Definition and descriptive statistics of variables.

Type	Content	Question stem and its assignment	Mean value	Standard deviation
Dependent variable				
Rights protection participation	Participate in online and offline collective rights protection actions	Political participation behavior: H1a. In the past 2 years, have you participated in the following events? Assignment: 1 = participated; 0 = Not participated in Political willingness to participate: H1b. If not, Are you willing to participate? Assignment: 1 = willing to participate; 0 = unwilling to participate	0.03(0.57)	0.181(0.495)
Social media participation	Reflect social issues to media such as newspapers, radio stations, and online forums		0.03(0.53)	0.157(0.499)
Contact-officer participation	Reflect opinions to government departments		0.07(0.58)	0.254(0.493)
Community participation	Participate in major decision-making discussions in the village		0.1(0.61)	0.298(0.487)
Election participation	Participate in village committee elections		0.37(0.61)	0.482(0.488)
Independent variable				
Overall social justice	Evaluation of the current overall social justice situation	1 to 10 points: 1 point represents "very unfair", 10 points represents "very fair"	6.68	2.222
Desert-based social justice	Wealth and income distribution; Jobs and employment opportunities	F4b1. What do you think is the level of justice in the following aspects of current social life? Assignment: 1 = Very unfair; 2 = Unfair; 3 = relatively fair; 4 = Very fair	2.967	0.903
Need-based social justice	Social security benefits such as public healthcare and elderly care		2.876	0.781
Equality-based social justice	Rights and treatment between urban and rural areas		3.21	0.853
	The actual political rights enjoyed by citizens; Judiciary and Law Enforcement			
Control variable				
Political identity	Party member = 1, others = 0		0.07	0.251
Annual household income	Logarithm of total income		10.481	1.272
Education level	Primary school and below = 1; Middle school = 2; High school = 3; Bachelor’s degree or above = 4		1.92	0.967
Subjective social status	Up = 1; Middle = 2; Down = 3		1.56	0.623
Internet usage	Use = 2; Not used = 1		1.41	0.492
Age	2019- Year of Birth		46.716	14.2
Gender	Male = 1; Female = 0		0.42	0.494
Cohort effect	After 40 = 1; After 50 = 2; After 60 = 3; After 70 = 4; Generation 80 = 5; Post 90s = 6		4.205	1.427
Regional effect	Northeast = 1; East China = 2; North China = 3; Central China = 4; South China = 5; Southwest = 6; Northwest = 7		3.56	1.340

Note: The data in parentheses represents political participation willingness, while the data outside parentheses represents political participation behavior.

Table 2 presents the results of the Logit model examining the relationship between overall social justice and political participation behavior among Chinese farmers. The coefficients indicate the impact of each variable on different forms of political participation. In Model 1, overall social justice demonstrates a positive but statistically insignificant association with rights protection participation. Model 2 reveals a significant positive correlation between overall social justice and election participation. Model 3 uncovers a highly significant positive effect of overall social justice on community participation. However, in Model 4, there is no statistically significant relationship between overall social justice and social media participation. Finally, Model 5 indicates a statistically significant negative correlation between overall social justice and contact-officer participation.The models also include additional variables. Apart from community participation, internet usage has a negative and statistically significant impact on all forms of political participation. Education level shows a positive and statistically significant relationship with rights protection participation and social media participation. Age, gender, subjective societal status, household income, political identity, cohort effects, and regional effects are also included as independent variables, exhibiting varying degrees of significance and directionality. Goodness-of-fit statistics indicate that these models explain a moderate variance in political participation behavior, with pseudo R-squared values ranging from 0.034 to 0.098. Numbers in parentheses represent standard errors. AIC represents Akaike Information Criterion, BIC represents Bayesian Information Criterion, and N represents the sample size used in the analysis.

**Table 2:** Logit model for overall social justice and political participation behavior of chinese farmers.

	(1)	(2)	(3)	(4)	(5)
	Rights protection participation	Election participation	Community participation	Social media participation	Contact-officer participation
Overall social justice	0.013	0.039***	0.1***	-0.063	-0.086***
	(0.033)	(0.012)	(0.020)	(0.040)	(0.022)
Internet usage	-0.166	-0.284***	-.301**	-0.150	-0.324**
	(0.181)	(0.068)	(0.020)	(0.237)	(0.122)
Education level	0.271***	0.022	0.003	0.451***	-0.052
	(0.085)	(0.036)	(0.058)	(0.099)	(0.065)
Age	0.024	0.026**	0.020	0.005	0.011
	(0.024)	(0.010)	(0.015)	(0.028)	(0.017)
Gender	0.457***	0.344***	0.509***	0.653***	0.658***
	(0.139)	(0.055)	(0.089)	(0.163)	(0.101)
Subjective social status	0.185*	0.021	0.287***	0.099	-0.0123
	(0.106)	(0.044)	(0.067)	(0.127)	(0.079)
Annual household income	0.216***	0.011	0.049	0.218**	0.076*
	(0.063)	(0.023)	(0.037)	(0.075)	(0.043)
Political identity	0.198	1.072***	1.494***	0.005	1.016***
	(0.223)	(0.111)	(0.120)	(0.277)	(0.144)
Cohort effect	-0.169	0.160*	0.113	-0.160	0.045
	(0.232)	(0.094)	(0.152)	(0.266)	(0.170)
Regional effect	0.109*	-0.074***	0.104***	0.013	0.150***
	(0.053)	(0.020)	(0.033)	(0.061)	(0.038)
N	6689	6689	6689	6689	6689
AIC	1981.117	8263.896	3970.245	1489.085	3310.437
BIC	2056.007	8338.787	4045.135	1563.975	3385.327
Pseudo R_2_	0.034	0.069	0.098	0.074	0.047

Note: The coefficient value is the result presented after processing the odds ratio value, representing the change in occurrence ratio for each unit of increase (decrease) in the independent variable; The values in parentheses are standard errors

***p<0.001

**p<0.01

*p<0.05

The explanation in the following table is the same.

As indicated by the models in Table 2, overall perception of societal justice does not exhibit statistically significant effects on rights protection participation. However, it shows a statistically significant negative effect on "Contact-officer participation," implying that weaker overall perception of societal justice among Chinese farmers increases the likelihood of engaging in individual activities related to contact-officer participation. Hence, this partially supports hypothesis 1.Furthermore, overall perception of societal justice demonstrates a positive and statistically significant impact on election participation and community participation behavior among Chinese farmers, thus validating hypothesis 2.

Table 3 presents the results of the Logit model examining the relationship between different perceptions of societal justice and political participation behavior among Chinese farmers. The coefficients represent the impact of each perception of societal justice on various forms of political participation. In Model 6, desert-based social justice exhibits a negative but statistically insignificant association with rights protection participation. In Model 7, it demonstrates a negative correlation with election participation. However, in Model 8, there is no significant relationship between desert-based social justice and community participation. Model 9 reveals a significant negative association between desert-based social justice and social media participation. Finally, in Model 10, desert-based social justice shows a statistically significant negative correlation with contact-officer participation.As shown in Model 7, equality-based perception of societal justice has a significant negative effect on rights protection participation. It does not show a significant association with either election participation or community participation (Models 6 and 8). However, in Model 9, equality-based social justice displays a significant negative relationship with social media participation. Furthermore, in Model 10, it exhibits a strong negative correlation with contact-officer participation. Across all five models, need-based social justice does not demonstrate statistically significant relationships with any form of political participation. Goodness-of-fit statistics indicate that these models explain a small to moderate portion of the variance in political participation behavior, with pseudo R-squared values ranging from 0.041 to 0.093.

**Table 3:** Logit model for different social justice perceptions and political participation behaviors of chinese farmers.

	(6)	(7)	(8)	(9)	(10)
	Rights protection participation	Election participation	Community participation	Social media participation	Contact-officer participation
Desert-based social justice	-0.122	-0.063	-0.041	-0.302*	-0.092
	(0.1)	(0.036)	(0.058)	(0.128)	(0.068)
Equality-based social justice	-0.282**	0.013	-0.009	-0.243*	-0.289***
	(0.098)	(0.036)	(0.06)	(0.121)	(0.067)
Need-based social justice	0.06	0.042	0.12	0.122	-0.137
	(0.114)	(0.041)	(0.066)	(0.143)	(0.077)
Internet usage	-0.113	-0.275***	-0.290**	-0.093	-0.203*
	(0.182)	(0.068)	(0.107)	(0.239)	(0.124)
Education level	0.259**	0.019	0.002	0.432***	-0.077
	(0.085)	(0.036)	(0.058)	(0.099)	(0.092)
Age	0.026**	0.027**	0.024	0.008	0.009
	(0.024)	(0.010)	(0.015)	(0.028)	(0.017)
Gender	0.447**	0.358***	0.538***	0.641	0.601***
	(0.139)	(0.055)	(0.089)	(0.164)	(0.102)
Subjective social status	0.221*	0.047	0.336***	0.108	-0.012
	(0.104)	(0.043)	(0.066)	(0.125)	(0.078)
Annual household income	0.204**	0.011	0.054	0.209**	0.055
	(0.063)	(0.023)	(0.037)	(0.076)	(0.043)
Political identity	0.222	1.089***	1.515***	-0.004	1.024***
	(0.223)	(0.111)	(0.120)	(0.277)	(0.145)
Cohort effect	-0.185	0.150	0.092	-0.185	0.061
	(0.233)	(0.094)	(0.152)	(0.267)	(0.171)
Regional effect	0.110*	-0.073**	0.106**	0.012	0.153***
	(0.053)	(0.020)	(0.033)	(0.061)	(0.037)
N	6689	6689	6689	6689	6689
AIC	1971.048	8275.237	3995.552	1481.296	3280.459
BIC	2059.555	8363.744	4084.059	1569.803	3368.966
Pseudo R_2_	0.041	0.068	0.093	0.081	0.056

According to Table 3, the indicator based on "Desert-based social justice" is statistically significant among Chinese farmers. This means that as farmers’ perception of desert-based social justice increases, their likelihood of engaging in social media participation also increases, thus validating Hypothesis 1.1. Additionally, the indicator based on "Equality-based social justice" demonstrates statistical significance in relation to rights protection participation, social media participation, and contact-officer participation. This implies that when farmers have a lower perception of equality-based social justice, they are more likely to engage in rights protection participation, social media participation, and contact-officer participation political activities, thus validating Hypothesis 1.3. However, there is no statistically significant relationship between the indicator for "Equality-based social justice" and other political participation behaviors such as election participation and community participation. Similarly, there is no statistically significant relationship between the indicator for "Need-based social justice" and any political participation behavior. Therefore, Hypotheses 1.2, 2.1, 2.2, and 2.3 are not supported. It can be observed that Hypothesis 1 is only partially validated for desert-based social justice, equality-based social justice, and need-based social justice. In other words, these three sub-indicators of perception of societal justice exhibit correlations with certain forms of participation behavior but do not apply to all participation behaviors.Specifically, desert-based social justice shows a negative correlation trend only with social media participation, indicating that when farmers have a lower perception of desert-based social justice, they are more likely to engage in social media activities. Furthermore, equality-based social justice exhibits a negative correlation trend with non-institutional political participation (such as mass movements, protests, etc.) and contact-officer participation. However, these three sub-indicators of perception of societal justice do not show any correlation trends with institutional political participation, such as election participation and community participation.

Table 4 presents the results of the Logit model examining the relationship between Chinese farmers’ perception of overall social justice and their willingness to engage in political participation. The coefficients represent the impact of perception of overall social justice on different forms of political participation. In Model 11, perception of overall social justice shows a statistically significant positive correlation with rights protection participation. In Model 12, it demonstrates a positive correlation with election participation, but only at a marginal significance level. Model 13 indicates a significant positive effect of perception of overall social justice on community participation. Similarly, in Model 14, perception of overall social justice has a highly significant positive impact on social media participation. Finally, in Model 15, perception of overall social justice exhibits a statistically significant positive correlation with contact-officer participation.Goodness-of-fit statistics indicate that these models explain a small to moderate portion of the variation in political participation willingness, with pseudo R-squared values ranging from 0.017 to 0.026.

**Table 4:** Logit model of overall social justice perception and political participation willingness of Chinese farmers.

	(11)	(12)	(13)	(14)	(15)
	Rights protection participation	Election participation	Community participation	Social media participation	Contact-officer participation
Overall social justice	0.032**	0.03*	0.037**	0.043***	0.034**
	(0.012)	(0.015)	(0.013)	(0.012)	(0.012)
Internet usage	-0.212**	-0.171	-0.277***	-0.196**	-0.218**
	(0.068)	(0.088)	(0.071)	(0.067)	(0.069)
Education level	0.157***	0.090*	0.168***	0.178***	0.154***
	(0.036)	(0.043)	(0.037)	(0.035)	(0.037)
Age	0.003	0.006	0.006	-0.003	-0.010
	(0.009)	(0.012)	(0.01)	(0.009)	(0.010)
Gender	0.149**	0.149*	0.286***	0.270***	0.273***
	(0.055)	(0.068)	(0.057)	(0.054)	(0.056)
Subjective social status	-0.019	0.073	0.071	-0.015	0.025
	(0.043)	(0.054)	(0.045)	(0.043)	(0.044)
Annual household income	-0.008	0.021	0.001	0.006	0.043
	(0.023)	(0.028)	(0.024)	(0.023)	(0.023)
Political identity	-0.087	-0.181	0.073	-0.125	0.052
	(0.110)	(0.176)	(0.133)	(0.107)	(0.119)
Cohort effect	-0.102	0.003	-0.020	-0.051	0.014
	(0.093)	(0.112)	(0.096)	(0.092)	(0.094)
Regional effect	0.1***	0.072**	0.098***	0.069***	0.078***
	(0.020)	(0.025)	(0.020)	(0.020)	(0.020)
N	6078	4047	5808	6139	5954
AIC	8145.277	5389.705	7625.803	8303.552	7884.523
BIC	8219.114	5459.068	7699.14	8377.499	7958.133
Pseudo R_2_	0.02	0.006	0.017	0.023	0.026

Table 5 presents the results of the Logit model examining the relationship between different perceptions of society justice among Chinese farmers and their willingness to engage in political participation. The coefficients represent the impact of each perception of social justice on various forms of political participation. In Model 16, desert-based perception of society justice shows no significant relationship with rights protection participation. Similarly, in Models 17 and 18, desert-based social justice exhibits no significant association with election participation or community participation, respectively. In Model 19, the relationship between desert-based social justice and social media participation is not statistically significant. Finally, in Model 20, desert-based social justice displays a non-significant negative correlation with contact-officer participation.As shown in Model 17, equality-based perception of society justice does not demonstrate a statistically significant relationship with rights protection participation. In Model 18, it has no significant association with election participation or community participation. Similarly, in Model 19, equality-based perception of society justice shows no significant relationship with social media participation. However, in Model 20, equality-based perception of society justice exhibits a positive correlation with contact-officer participation, albeit not statistically significant.As indicated in Model 18, need-based perception of society justice has no significant relationship with rights protection participation, election participation, or community participation. Additionally, in Models 19 and 20, need-based social justice does not show any statistically significant associations with social media participation or contact-officer participation.Goodness-of-fit statistics indicate that these models explain a small to moderate portion of the variation in political participation willingness, with pseudo R-squared values ranging from 0.016 to 0.026.

**Table 5:** Logit model of different perceptions of social justice and political participation willingness of Chinese farmers.

	(16)	(17)	(18)	(19)	(20)
	Rights protection participation	Election participation	Community participation	Social media participation	Contact-officer participation
Desert-based social justice	-0.043	0.003	-0.011	-0.018	-0.042
	(0.036)	(0.045)	(0.037)	(0.036)	(0.037)
Equality-based social justice	-0.017	0.046	-0.015	-0.032	0.031
	(0.037)	(0.044)	(0.038)	(0.037)	(0.038)
Need-based social justice	-0.038	-0.076	-0.048	-0.033	-0.078
	(0.042)	(0.053)	(0.043)	(0.042)	(0.042)
Internet usage	-0.188**	-0.155	-0.254***	-0.174*	-0.192**
	(0.068)	(0.088)	(0.072)	(0.067)	(0.069)
Education level	0.152***	0.091*	0.165***	0.175***	0.149***
	(0.036)	(0.043)	(0.037)	(0.035)	(0.037)
Age	0.003	0.006	0.007	-0.002	-0.010
	(0.009)	(0.012)	(0.010)	(0.009)	(0.010)
Gender	0.153**	0.155*	0.290***	0.276***	0.281***
	(0.055)	(0.068)	(0.057)	(0.054)	(0.056)
Subjective social status	0.009	0.093	0.098*	0.020	0.056
	(0.043)	(0.053)	(0.045)	(0.042)	(0.044)
Annual household income	-0.009	0.022	0.000	0.005	0.051
	(0.023)	(0.028)	(0.024)	(0.023)	(0.044)
Political identity	-0.067	-0.158	0.088	-0.103	0.041
	(0.110)	(0.176)	(0.133)	(0.107)	(0.023)
Cohort effect	-0.105	-0.002	-0.022	-0.055	0.011
	(0.093)	(0.112)	(0.096)	(0.091)	(0.094)
Regional effect	0.102***	0.072**	0.101***	0.071***	0.080***
	(0.020)	(0.025)	(0.020)	(0.020)	(0.020)
N	6078	4047	5808	6139	5954
AIC	8150.21	5395.288	7635.11	8316.621	7888.622
BIC	8237.472	5477.262	7721.781	8404.013	7975.616
Pseudo R_2_	0.019	0.006	0.016	0.022	0.026

Perception of overall social justice shows a positive and statistically significant relationship with willingness to participate in election and community activities. In other words, when farmers perceive a greater sense of overall social justice, they are more inclined to engage in election participation or community participation. However, the effect of perception of overall social justice on political participation willingness related to contact-officer participation among Chinese farmers presents an opposite scenario, exhibiting a positive and statistically significant effect. This partially confirms Hypothesis 3, which suggests that individuals who do not participate in political activities are more willing to engage in such activities as they perceive greater fairness in overall social justice.Furthermore, according to the statistical results presented in Table 5, there are no significant relationships between desert-based social justice, equality-based social justice, need-based social justice, and willingness to engage in political participation. Consequently, Hypotheses 3.1, 3.2, and 3.3 have not been supported.

## Discussion

### Perceptions of social justice and political participation

Generally speaking, there is a distinction between macro-level justice and micro-level justice. The assessment of macro-level justice is particularly susceptible to ideological and political beliefs. It tends to be guided by utopian rather than existentialist standards of justice [84]. On the other hand, micro-level justice focuses on individuals and their beliefs about the fairness of outcomes, making it more pragmatic in nature. Therefore, in this study, we examine perception of overall societal justice separately from various subtypes of perception of societal justice.Therefore, in this study, we separate the perception of overall social justice from various subtypes of perception of society justice. The former represents individuals’ overall perception of the level of justice in the current society, while the latter is more closely related to the actual perceptions associated with personal experiences.

The results of this study indicate that, for non-institutional political participation, the perception of overall social justice does not have a statistically significant effect on rights protection participation. However, it exhibits a negative statistically significant effect on "contact-officer participation," meaning that the weaker the perception of overall social justice among Chinese farmers, the higher the likelihood of engaging in individual activities related to contact officers. The perception of overall social justice has a positive statistically significant effect on election participation and community participation among Chinese farmers. On the other hand, the three sub-indicators of perception of society justice do not show any correlational trends with institutionalized political participation such as election participation and community participation.This phenomenon stems from the "Good Official" complex in traditional Chinese culture [85,86]. When individuals perceive unfair treatment, they are more inclined to seek morally upright individual officials to uphold justice on their behalf, rather than resorting to formal institutional mechanisms such as courts or arbitration, as Westerners typically do. The perception of overall social justice, as an underlying and universal belief, is closely tied to people’s trust in the government. In China, political trust in the government follows an incremental pattern from local to central levels. Based on this political trust, the perception of overall social justice drives individuals to engage in institutionalized political participation. This may be associated with a "Shengjunqingjie(sage ruler complex)" [87] prevalent in traditional Chinese culture, where there is a tendency to direct anger towards lower-ranking officials perceived as morally corrupt when facing personal experiences of unfair treatment, rather than attributing it to the authority of the central government or the formal political system. Consequently, different sub-indicators of perception of society justice do not influence people’s engagement in institutionalized political participation activities.

In the case where economic productivity is primarily focused on cooperation, Desert becomes the main criterion for social justice. When the establishment or maintenance of social relationships itself is the primary objective, Equality serves as the main standard. Lastly, when individual development and well-being are the primary goals of cooperation, Need becomes the principal criterion. Our research findings indicate a negative correlation between Desert-based social justice and social media participation, suggesting that farmers are more likely to engage in social media when they have lower perceptions of Desert-based social justice. In situations where economic productivity and cooperation are the primary objectives, Desert-based social justice becomes the main standard. This implies that individuals prioritize resource and reward distribution based on personal contributions, believing that everyone should receive what they deserve. Therefore, when Chinese farmers evaluate Desert-based social justice negatively, they may be more inclined to pursue a sense of fairness through engaging in media activities, hoping to improve social justice by conveying information and expressing their voices. In China, private enterprises and private capital are often criticized by state media. In such circumstances, seeking exposure through state media or forums to address issues appears to be a more rational and practical choice for the public. Equality-based social justice exhibits a negative correlation with non-institutional political participation, such as rights protection participation, social media participation, and contact-officer participation. When Equality-based social justice becomes the primary criterion, it signifies a greater emphasis on equality and fairness, highlighting that everyone should have equal rights and opportunities. This suggests that the non-institutional political participation of Chinese farmers is more likely driven by their perception of unequal political rights and opportunities compared to others. Given that contacts with officials often involve power and privilege disparities, Equality-based social justice also demonstrates a negative correlation with contact-officer participation. Finally, when Need-based social justice becomes the main criterion, it indicates a focus on meeting basic needs and providing necessary support to foster individual development and well-being. The Need-based social justice indicator does not exhibit a direct correlation with any type of political participation. This may stem from the traditional sense of justice held by Chinese farmers, who generally believe that meeting basic needs and providing necessary support are not related to politics and are not the government’s responsibility.

### Political participation and political participation willingness

We believe that political participation by the public is often influenced by multiple factors, driven by both emotional and rational considerations. On one hand, individuals who hold stronger beliefs in social justice, as per the relative deprivation theory and the belief in a just world hypothesis, are more likely to engage in institutional political activities actively. Moreover, as individuals perceive greater unfairness, their likelihood of participating in "non-institutional political activities" also increases. On the other hand, factors such as an immature governance model, high costs of legitimate bargaining, the influence of state power on daily politics, and deficiencies in the overall social governance system can contribute to public engagement in non-institutional political activities. Conversely, if the public perceives society as fair, their likelihood of choosing institutionalized avenues for participation increases. In other words, the improvement in political institutions encourages citizens to actively engage in political life.

Emotional and rational factors coexist within the behavior choices and preferences of individuals in political participation, with their effects and sequences determined by the behaviors and perceptions of individuals in different societal contexts. Drawing on a nationwide database, this study conducts a comparative analysis of behavioral models and willingness models of political participation to gain a clearer understanding of the intricate relationship and underlying logic between the perception of societal justice and political participation. It is revealed that due to high risks and costs, individuals engage in non-institutional political activities only when emotional accumulation reaches a certain threshold. While the term "political participation" typically encompasses activities such as voting, running for office, demonstrations, and riots, these modes of participation exhibit significant variations across different political systems, cultures, and time periods. Political participation among Chinese farmers fundamentally differs from that in more open societies. The presence of a powerful government and party institutions, extensive penetration of the state into society, a lack of independent advocacy groups and other mechanisms, constraints on mass media, and a prolonged absence of freedom and competitive elections shape distinct meanings for participation [88]. In China, rights protection behaviors like parades and demonstrations are strictly prohibited by the government [89], contact-officer participation often involves illicit trade of power for money or bribery [90], and social media participation faces stringent network censorship, potentially resulting in post deletion and account suspension penalties [91]. Thus, from the perspective of risks and costs, in China, non-institutional political activities carry greater weight than institutional political activities, with rights protection surpassing contact-officer participation and social media participation.

**Fig 1:**
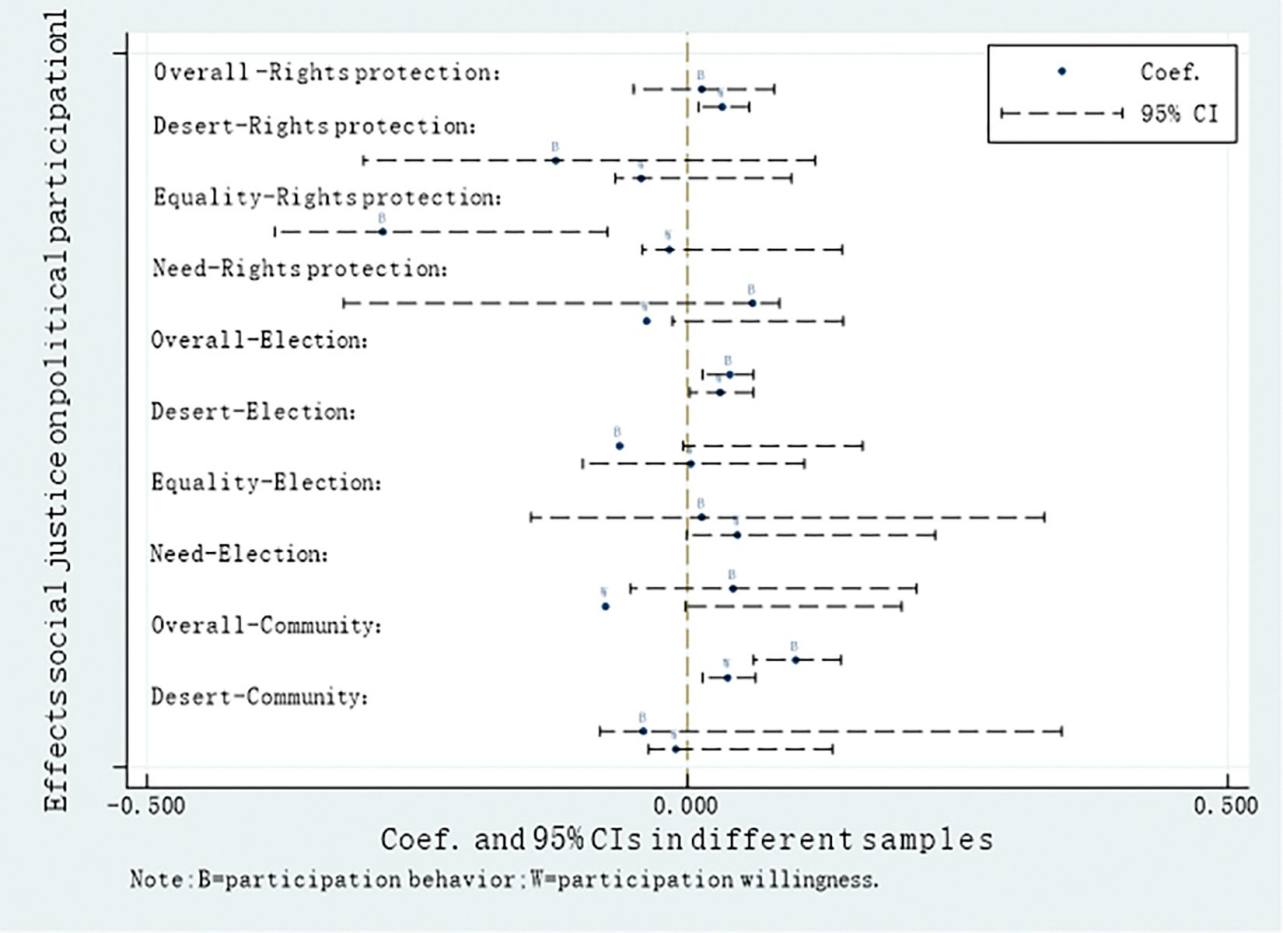
Effects social justice on political participation(1).

**Fig 2:**
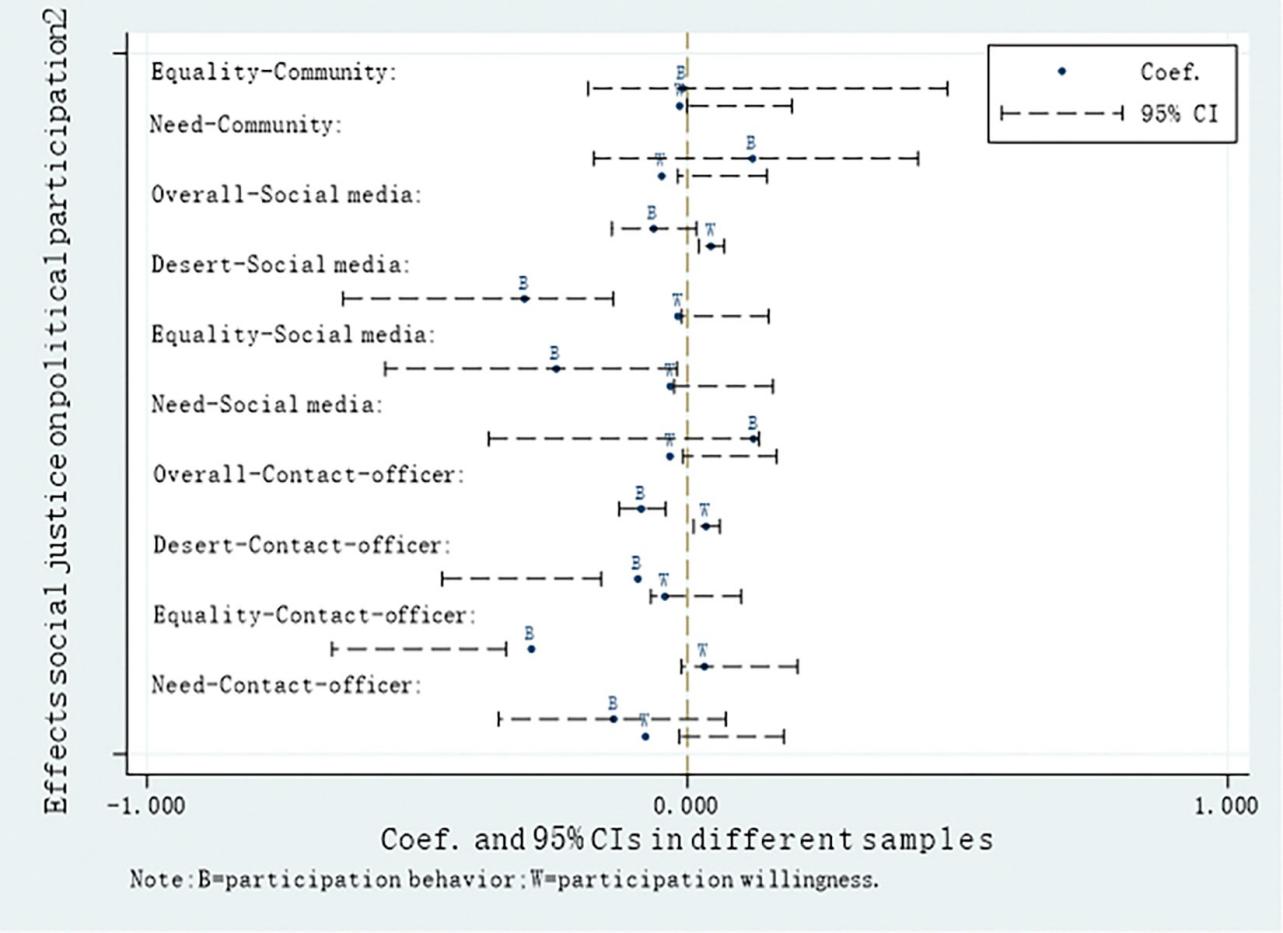
Effects social justice on political participation(2).

Figs 1 and 2 have been meticulously designed to provide a visually compelling and unambiguous depiction of how different types of perception of societal justice influence diverse forms of political participation, highlighting the disparities between "behavior" and "willingness."The results of this study indicate that Overall social justice has a statistically significant negative effect on Contact-officer participation, while Desert-based social justice has a statistically significant negative effect on Social media participation. Equality-based social justice exhibits a statistically significant negative relationship with non-institutional political participation behaviors such as Rights protection, Contact-officer participation, and Social media participation. Additionally, the perception of Overall social justice in relation to the willingness model of political participation among Chinese farmers and Contact-officer participation yields results opposite to those of the behavior model, further validating the previous findings. If Chinese farmers who choose Contact-officer participation are influenced by rational factors, then non-participating individuals are influenced by emotional factors. As mentioned earlier in this paper, traditional Chinese culture embodies an "Ideal Official" narrative, where people seek morally upright officials to uphold justice when they perceive unfair treatment. However, this is an idealized emotional appeal, as truly "Good Officials" are often scarce in reality, with many officials potentially being corrupt. Therefore, when Chinese farmers perceive overall societal justice, they tend not to choose Contact-officer participation because this behavior often entails significant costs in terms of financial and social capital investment.

### Climate change and political participation

In the realm of policy responses to climate change, contemporary democratic countries typically promote public participation, aiming to involve a diverse range of stakeholders in decision-making processes [92,93]. Consequently, citizens’ perception of societal justice regarding climate change risks is closely intertwined with the outcomes of institutional political participation. For instance, in India, the public’s perception of societal justice concerning climate change holds a certain degree of influence over the results of specific periods of political elections [94]. In line with this, within a democratic system, obstacles to implementing climate change-related policies often arise from individuals’ personal perception of "environmental justice." For example, in the United States, many citizens express skepticism towards the dire consequences of climate change proclaimed by the media and experts [95]. However, in China, issues and policies related to climate change and environmental justice are typically decided by a select group of experts and bureaucratic elites, enforced by the state. Despite the existence of formal and institutional channels for political participation, such as the Local People’s Congress and village committees, which are legally mandated to exercise significant decision-making power and facilitate open and extensive discussions on major livelihood issues, under an authoritarian regime, the scope of non-institutional political participation among Chinese farmers is severely restricted. Although contrasting the views of the American public, Chinese citizens generally acknowledge the reality of "global warming" and support environmental justice measures at the national level [96]. Nevertheless, when they perceive personal interests being deprived or injustices being committed, they resort to alternative forms of participation beyond the established institutional channels.

Using the example of the "Coal to gas" policy project implemented by the Chinese government, China adopted this policy in certain rural areas in the northern regions to address the increasingly severe issue of climate change. The policy aimed to promote increased consumption of natural gas while reducing coal consumption [97]. However, within less than a year of its implementation, the "Coal to gas" project was urgently halted by the national environmental protection department.The fundamental reason behind this decision lies in China’s context, where coal has undergone complete marketization, but natural gas has not achieved full marketization. As a result, pricing authority remains in the hands of the state and state-owned enterprises. The state intervenes in people’s preferences by narrowing pricing authority, which leads some individuals to feel their rights being deprived. When these deprived individuals discover the resulting unfairness caused by such deprivation, political resistance occurs.Moreover, it is crucial to note that the correction of this unfairness is achieved through non-institutional forms of political participation, such as engagement with contact officers, exposure on social media platforms, and even political resistance, rather than relying solely on institutional political participation channels established by the state.

## Conclusion

Using data from the CSS 2019 survey, this study examines the relationship between Chinese farmers’ perception of societal justice and political participation from multiple perspectives. We discovered that Chinese farmers’ perception of overall societal justice exhibits a U-shaped relationship with various forms of political participation. Specifically, it shows a significant negative correlation with non-institutional political participation, such as contact-officer participation, but a significant positive correlation with institutional political participation types like community participation and election participation.Our further research indicates that the three subtypes of perception of societal justice are significantly negatively correlated only with non-institutional political participation, while their statistical relationship with institutional political participation is not significant. We believe that the underlying reason for this phenomenon lies in the unique interpretation of societal justice within Chinese traditional culture. Additionally, through a comparative analysis of models on political participation behavior and willingness, we found that despite significant inequalities and disparities in institutional structures and levels of economic development between rural and urban areas in China, rational considerations of the risks and costs associated with defying the government deter Chinese farmers from engaging in non-institutional politic activities unless their emotional resentment towards unjust practices reaches a certain threshold.

Compared to non-institutional political participation, it is undeniable that institutional political participation has a positive impact on enhancing public trust in politics and resolving conflicts between the government and its citizens. Therefore, based on our research findings, we propose the following policy recommendations:

Firstly, due to a significant negative correlation between Chinese farmers’ perception of overall social justice and non-institutional political participation, but a significant positive correlation with institutional political participation, which is often associated with macro-level political ideologies, it is necessary for the Chinese government to continue promoting their governing philosophy regarding social justice to maintain societal harmony and mitigate the negative societal impacts brought by non-institutional political participation. This includes shaping a positive image of their commitment to social justice.Furthermore, the three subtypes of societal justice perceptions related to "Desert," "Equality," and "Need" show a significant negative correlation with non-institutional political participation, but their statistical relationship with institutional political participation is not significant. In other words, when individuals encounter perceived injustice at the individual benefit level, such as in "Desert," "Equality," and "Need," they are more inclined to engage in non-institutional political participation rather than institutional political participation. Therefore, it is necessary for the Chinese government to grant substantive power to institutional political participation, such as community participation and election participation, making them effective mechanisms for citizens to seek social justice and protect individual rights, rather than merely symbolic displays of state authority legitimacy.Thirdly, due to the coexistence of emotional and rational factors in the willingness and behavior of Chinese farmers’ political participation, the government needs to simultaneously address citizens’ emotional and rational needs rather than waiting for their sense of deprivation and anger towards injustice to reach a breaking point before implementing remedial measures. At times, despite deep feelings of injustice, individuals may choose silence and endurance due to the rational costs and consequences associated with non-institutional political participation. However, the accumulation and eruption of such endurance can lead to severe societal consequences.

Finally, our research has both strengths and limitations. Existing studies in this field often focus on the singular aspect of public perception of societal justice while overlooking the variations in individuals’ perceptions at macro and micro levels. Furthermore, the existing literature typically emphasizes the influence of either emotional or rational factors on individuals’ willingness and outcomes of political participation, disregarding the fact that emotions and rationality often coexist in reality. Our study offers a more comprehensive perspective, addressing the aforementioned limitations in existing literature and research.Nevertheless, it is important to acknowledge the potential limitations of this study. Firstly, this study used cross-sectional data which only provides observations at a single time point or within a specific time period. As a result, it cannot establish the time sequence between variables.Secondly, categorizing Political participation in the Chinese context as institutionalized and non-institutionalized forms may not accurately reflect the diversity and complexity of political participation behaviors. Thirdly, the variables studied in this research were established within a specific Chinese context, and the conclusions and findings may only be applicable to this specific cultural and social background, potentially limiting external validity. In conclusion, researchers must pay close attention to these limitations when interpreting their research results, ensuring that they are generalizable and applicable to a wider range of contexts.
